# Historical Evolution of Second-Line Therapy in Non-Small Cell Lung Cancer

**DOI:** 10.3389/fmed.2017.00004

**Published:** 2017-01-23

**Authors:** Chiara Lazzari, Alessandra Bulotta, Monika Ducceschi, Maria Grazia Viganò, Elena Brioschi, Francesca Corti, Luca Gianni, Vanesa Gregorc

**Affiliations:** ^1^Department of Oncology, Division of Experimental Oncology, IRCCS San Raffaele Scientific Institute, Milan, Italy

**Keywords:** non-small cell lung cancer, second line, docetaxel, pemetrexed, erlotinib, angiogenesis, immunotherapy

## Abstract

Innovative therapeutic agents have significantly improved outcome with an acceptable safety profile in a substantial proportion of non-small cell lung cancer (NSCLC) patients, who depend on oncogenic molecular alterations for their malignant phenotype. Despite the survival improvement achieved with first-line chemotherapy, about 30% of patients do not obtain a tumor response. Moreover, those patients, initially sensitive to treatment, acquire resistance and develop tumor progression after a median of about 5 months. Approximately 60% of the patients progressing from first-line chemotherapy receive further systemic treatment in the second-line setting. Moreover, new options have emerged in the second-line armamentarium for the treatment of patients with NSCLC, including immune checkpoint inhibitors and antiangiogenic agents. The current review provides an overview on the clinical studies that gained the approval of chemotherapy agents (*docetaxel and pemetrexed*) and epidermal growth factor receptor gene–tyrosine kinase inhibitors as second-line treatment options for NSCLC patients, not carrying molecular alterations.

## Introduction

Lung cancer is the leading cause of cancer death in the world (1) and non-small cell lung cancer (NSCLC) accounts for approximately 85% of cases. The majority of patients are diagnosed with advanced or metastatic disease. Despite the progresses in the treatment of NSCLC, the prognosis remains poor, with an estimated 5 years overall survival (OS) of only 16%.

For a long time, platinum doublet chemotherapy has been the standard first-line treatment option for NSCLC patients (2, 3). Until 2005, treatment choice was mainly based on the distinction between NSCLC and small cell lung cancer. The approval of bevacizumab in 2006 (4, 5) and pemetrexed in 2008 (6) raised the issue that discriminating between squamous and non-squamous histology was a crucial element for therapeutic selection, since bevacizumab and pemetrexed can be administered to patients with non-squamous tumors only, for safety and efficacy reasons.

During the past 10 years, thanks to the technological advances, our knowledge on NSCLC tumor biology has improved (7). Different driver molecular alterations, responsible for the development of oncogene-addicted NSCLC tumors, have been identified, especially in the subgroup of patients with adenocarcinoma (8–12). Currently, NSCLC is not considered a single homogenous entity, but as a heterogeneous disease, including rare molecularly classified lung tumors, that are susceptible to targeted inhibition (13–17). Patients who carry activating mutations in the epidermal growth factor receptor gene (EGFR) or translocations in the anaplastic lymphoma kinase gene are treated with their specific tyrosine kinase inhibitors (TKIs), while platinum-based doublet chemotherapy with or without bevacizumab remains the first-line standard of care for patients in whom no molecular alteration is identified.

Despite the survival improvement achieved with first-line chemotherapy (18), about 30% of patients do not obtain a tumor response. Moreover, those patients, initially sensitive to treatment, acquire resistance and develop tumor progression after a median of about 5 months (19). Approximately 60% of the patients progressing from first-line chemotherapy receive further systemic treatment in the second-line setting. Currently, second-line therapy is based on docetaxel, pemetrexed, erlotinib, nivolumab, or the combination of docetaxel with nintedanib or ramucirumab. The current review provides an overview on the clinical studies that gained the approval of chemotherapy agents and EGFR–TKIs as second-line treatment options for NSCLC patients, not carrying molecular alterations.

## Docetaxel and Pemetrexed

The TAX317 study (20) was the first phase III trial showing a survival advantage of second-line chemotherapy in NSCLC patients, previously treated with platinum-based regimen (Table 1). One hundred three patients, stratified according to Eastern Cooperative Oncology Group performance status (ECOG PS) and best response to first-line chemotherapy, were randomized between two different doses of docetaxel (100 and 75 mg/m^2^) and best supportive care. Docetaxel was associated with significantly longer OS and time to progression (TTP), compared with best supportive care. The advantage was significantly greater in the group receiving docetaxel at the dose of 75 mg/m^2^, probably due to the higher frequency of febrile neutropenia and deaths observed in patients under treatment with 100 mg/m^2^. These results were confirmed by the phase III TAX320 study (Table 1), which compared docetaxel at the dose of 100 or 75 mg/m^2^, with vinorelbine or ifosfamide in 373 NSCLC patients, who had previously failed platinum-containing chemotherapy (21). Docetaxel was associated with longer TTP and progression-free survival (PFS). Even though OS did not differ between the three regimens, a significant greater percentage of patients receiving docetaxel at the dose of 75 mg/m^2^ was alive during the first year, compared with those randomized in the vinorelbine or ifosfamide arms (Table 1). Based on these data, docetaxel at the dose of 75 mg/m^2^ has become the reference control arm for second-line chemotherapy for patients with advanced NSCLC.

**Table 1:** **Clinical trials exploring second-line chemotherapy**.

Study	Treatment	*N* of pts	Major toxicities	Progression-free survival HR (95%CI)	Overall survival HR (95% CI)
TAX317 (20)	Docetaxel (100 or 75 mg/m^2^) vs best supportive care	103	Leukopenia, neutropenia, hair loss	–	*p* = 0.01
TAX320 (21)	Docetaxel (100 or 75 mg/m^2^) vs vinorelbine or ifosfamide	373	Leukopenia, neutropenia, hair loss for docetaxel	*p* = 0.005	5.5 vs 5.7 m (NS)
DISTAL-01 (22)	Docetaxel (75 mg/m^2^ q21) vs docetaxel (33 mg/m^2^ weekly)		Weekly: non-neutropenic infection	–	HR 1.04 (0.77–1.39) *p* = 0.80
			3-weekly: leukopenia, neutropenia, hair loss		
JMEI (23)	Pemetrexed (500 mg/m^2^) vs docetaxel (75 mg/m^2^)		Leukopenia, neutropenia, hair loss for docetaxel	HR 0.97 (0.82–1.16)	HR 0.99 (0.8–1.20)

*NS, not significant; HR, hazard ratio*.

With the aim to reduce the frequency of grade 3–4 hematologic adverse events, observed in a high proportion of the patients enrolled in the TAX317 and TAX 320 trials (54 and 67%, respectively) (20, 21), two docetaxel schedules (75 mg/m^2^ administered every 3 weeks and 33.3 mg/m^2^ administered weekly) were investigated in the phase III DISTAL-1 study (Table 1) (22). No significant difference was observed in terms of OS or global quality of Life (QoL), even though the weekly docetaxel resulted in significantly lower incidence of leukopenia, neutropenia, and hair loss, but higher occurrence of non-neutropenic infections. Moreover, an improvement in some of the QoL items, such as pain and cough, were reported with the weekly regimen (Table 1). In order to better compare the efficacy and the safety profile of the weekly and three-weekly docetaxel regimens, an individual patient data meta-analysis, including three phase III and two phase II randomized trials, enrolling 865 patients, was performed (24). No difference in terms of OS or objective response rate (ORR) was found, but a significant advantage in terms of severe and febrile neutropenia was confirmed in favor of the weekly schedule, thus suggesting that weekly docetaxel represents a valid alternative to the three-weekly administration (Table 1).

Another therapeutic opportunity in the second-line setting is represented by the antifolate pemetrexed (25). Based on the results of a phase III trial, showing the non-inferiority of pemetrexed in terms of PFS, OS, and ORR and a more favorable toxicity profile over docetaxel, with fewer grade 3–4 neutropenia and febrile neutropenia (23), in 2004, pemetrexed was approved in the USA and Europe for the second-line treatment of patients with advanced NSCLC (Table 1). A previous retrospective analysis, focusing on the toxicities observed in 246 patients treated between 1995 and 1999 with pemetrexed, indicated that high pretreatment plasma homocysteine levels were associated with severe toxicity. This finding suggested that decreasing homocysteine levels, through the use of folate and vitamin B12 supplementation, would have improved pemetrexed safety profile without decreasing its efficacy (26). The favorable toxicity profile of pemetrexed was confirmed in the subset analysis performed in 86 out of 571 patients with ≥70 years, enrolled in the phase III registration trial (27). A following phase III study, exploring cisplatin–pemetrexed as a first-line option, showed that pemetrexed is more effective in patients with non-squamous histology, due to the low expression of thymidylate synthase, a gene involved in the synthesis of folate and responsible for pemetrexed resistance in patients with lung squamous tumors (6, 28). Accordingly, the second-line indication for pemetrexed was revised to include patients with advanced non-squamous histology only.

In order to improve the therapeutic options, several trials have explored the efficacy and safety of doublet chemotherapy. An individual patient data analysis, including 847 patients, enrolled in six randomized trials (four phase II and two phase III), comparing mono-chemotherapy with doublet chemotherapy, was performed (29). Even though there was a statistically significant PFS improvement (of about 2 weeks) and a double RR with combination regimen, no survival prolongation was observed. These findings do not appear clinically relevant, and mono-chemotherapy has remained the standard of care for second-line treatment.

## Chemotherapy or EGFR–TKIs in Second-Line Setting

In 2005, the phase III BR.21 trial (Table 2) compared the efficacy of the EGFR–TKI erlotinib with best supportive care in previously treated 731 advanced NSCLC patients, with ECOG PS 0-3. Significant improvement in terms of OS, PFS, and QoL was observed in the erlotinib arm (30). For a long time, the identification of molecular and clinical features, able to predict which patients could benefit more from EGFR–TKIs, has been the focus of much research. Based on the clinical data from patients, enrolled in the BR.21 trial, who early progressed (<8 weeks), or died (within 3 months from randomization) under erlotinib, a prognostic score, including 10 factors (smoking history, ECOG PS, weight loss, anemia, lactic dehydrogenase, response to prior chemotherapy, time from diagnosis, number of prior regimens, EGFR copy, and ethnicity), was built (31). Only 10% of the patients were classified in the low risk group and had high significant survival advantage with erlotinib over placebo. Moreover, the retrospective analysis of Kirsten rat sarcoma viral oncogene homolog, EGFR mutations, and EGFR gene copy number by fluorescence *in situ* hybridization (FISH) showed that EGFR mutations and high copy number were predictive of response to erlotinib, but only EGFR FISH resulted as a significant predictive marker of differential survival benefit (32). Despite EGFR activating mutations being identified in 2004 (8, 33), their role, as predictive biomarkers of sensitivity to EGFR–TKIs, was recognized only in 2009, following the results from the phase III IPASS study. The study reported a significant PFS and ORR advantage of gefitinib over platinum-based first-line chemotherapy in EGFR mutant patients and a detrimental effect in the EGFR wild-type subgroup (34). These findings shifted the development of EGFR–TKIs toward the first-line treatment of EGFR oncogene-addicted tumors and raised the question if erlotinib was an appropriate therapeutic option for EGFR wild-type patients or patients with unknown molecular status in the second-line setting. Other considerations include understanding the differences between QoL and toxicity profile for EGFR–TKIs in comparison to standard of care in second-line and beyond.

**Table 2:** **Clinical trials exploring epidermal growth factor receptor gene–tyrosine kinase inhibitors with second-line chemotherapy**.

Study	Treatment	*N* of pts	Major toxicities	Progression-free survival HR (95%CI)	Overall survival HR (95% CI)
BR.21 (30)	Erlotinib vs best supportive care	731	Skin rash, diarrhea	HR 0.61 (0.51–0.74) *p* < 0.001	HR 0.70 (0.58–0.85) *p* < 0.001
TITAN (35)	Erlotinib vs docetaxel or pemetrexed	424	Skin rash, diarrhea for erlotinib	–	HR 0.96 (0.78–1.19) *p* = 0.73
DELTA (36)	Erlotinib vs docetaxel	301		HR 1.22 (0.97–1.55) *p* = 0.09	HR 0.91 (0.68–1.22) *p* = 0.53
TAILOR (37)	Docetaxel vs erlotinib	222	Leukopenia, neutropenia, hair loss for docetaxel	HR 0.71 (0.53–0.95) *p* = 0.02	HR 0.73 (0.53–1.0) *p* = 0.05
PROSE (38)	Docetaxel or pemetrexed vs erlotinib	285		HR = 1.35 (1.05–1.73) *p* = 0.020	HR = 1.22 (0.93–1.59) *p* = 0.148
HORG (39)	Erlotinib vs pemetrexed	322	Skin rash, diarrhea for erlotinib	*p* = 0.136	*p* = 0.986
LUX-LUNG 8 (40)	Afatinib vs erlotinib	795	Skin rash, diarrhea	HR 0.82 (0.68–1.0) *p* = 0.04	HR 0.81 (0.69–0.95) *p* = 0.01

Erlotinib has advantages in terms of toxicity, route of administration, and QoL. Its efficacy was compared with docetaxel and pemetrexed in different Phase III trials. Even though these studies had a different statistical design, the results were similar.

The TITAN trial (Table 2) was designed to demonstrate a 25% improvement in median OS of erlotinib vs chemotherapy (docetaxel and pemetrexed) in 648 unselected NSCLC patients, who had progressed during first-line platinum doublet chemotherapy (35). Due to the slow accrual, the trial was prematurely closed, enrolling 424 patients only. No significant difference in terms of OS or PFS was seen between erlotinib and chemotherapy. Tumor samples were mandatory to enter the trial, and EGFR mutation status was available in 160 of the enrolled patients. Comparable OS and PFS were observed in the EGFR wild-type subgroup under chemotherapy or erlotinib.

These results were partly confirmed by the DELTA and the HORG studies (Table 2). The primary objective of the DELTA trial was to show 1-month PFS superiority of erlotinib over docetaxel in unselected second- or third-line 301 Asian NSCLC patients (36). Even though no significant difference was observed in terms of PFS and OS, docetaxel statistically prolonged PFS in the EGFR wild-type subgroup (199 patients out of 255 analyzed). However, this improvement did not translate into longer survival. The HORG study randomized 322 NSCLC patients, previously progressed to one or two chemotherapy lines, between erlotinib and pemetrexed (39). Squamous histology was not an exclusion criterion and the primary end-point was TTP. There was no difference in terms of TTP, ORR, or OS between the two treatment arms. EGFR mutations were analyzed in 123 patients, and no OS, TTP, or ORR difference was observed, but EGFR wild-type patients had higher disease control rate under pemetrexed over erlotinib.

In contrast with the other studies, significantly longer PFS and OS (at the adjusted multivariate analysis) were found in favor of docetaxel in the 222 EGFR wild-type patients, enrolled in the TAILOR trial (Table 2), whose primary objective was to show 14% OS improvement at 1 year of docetaxel over erlotinib in EGFR wild-type NSCLC patients (37). The cross-over treatment in further lines was not allowed and only taxane-naïve patients were included. These differences might have influenced the OS results.

Finally, the PROSE study (Table 2) randomized 285 unselected second-line NSCLC patients, who were blinded classified according to a serum proteomic algorithm (the VeriStrat^®^ test), previously developed, with the aim to identify patients who could benefit from EGFR–TKIs (38, 41). Patients were stratified by the proteomic algorithm, ECOG PS, and smoking history. The primary end-point was OS, and the primary hypothesis was to demonstrate the existence of a significant interaction between the proteomic classification and treatment efficacy. The VeriStrat^®^ test is a multivariate biomarker, developed using eight *m/z* ratio mass spectrometric peaks. It classifies patients into two groups (*good* and *poor*), according to the clinical outcome observed under treatment with EGFR–TKIs. The results from the PROSE study were comparable to previous reports and showed that the PFS was longer in patients receiving chemotherapy, while no OS difference was found in the intent to treat analysis. The VeriStrat^®^ test was prognostic, since g*ood* classified patients had better OS and PFS than *poor* classified ones. Furthermore, while *good*-classified patients derived similar OS benefit from erlotinib and chemotherapy, VeriStrat *poor*-patients had significantly shorter OS under erlotinib, suggesting that the algorithm was also predictive of differential OS benefit between erlotinib and chemotherapy. EGFR mutations were analyzed in 176 patients included in the primary analysis, 14 of whom carried EGFR mutations. No statistical significant interaction was observed between VeriStrat classification and the EGFR mutation status, and comparable PFS and OS results were found in the EGFR wild-type subgroup. The prognostic and predictive role of the VeriStrat^®^ test was also retrospectively evaluated in 441 patients from the BR.21 trial (42). VeriStrat results demonstrated prognostic for OS and PFS, and predictive of response, but not predictive of differential benefit from erlotinib vs placebo.

Several meta-analyses have been performed to address the issue about the efficacy of EGFR–TKIs or chemotherapy in the second-line setting for the treatment of EGFR wild-type patients or patients with unknown molecular status. Recently, a meta-analysis, including 10 randomized trials and 1,119 EGFR wild-type patients, showed a significant PFS improvement for chemotherapy compared with EGFR–TKI therapy, with no OS difference (43). These results were confirmed by an individual patient data analysis, not yet published, and presented at ASCO in 2015, including 587 EGFR wild-type patients, enrolled in TAIOLR, DELTA, and PROSE studies. Chemotherapy determined longer PFS, which did not translate into longer OS.

Based on these findings, there are sufficient evidences suggesting that, in EGFR wild-type patients with good ECOG PS, chemotherapy determines a greater disease control, although with more toxicity and without increasing survival.

Results from PROSE might partly explain as to which factors can contribute to the discrepancy observed between PFS and OS. One possible explanation is that, since *poor* classified patients have a detrimental effect under erlotinib, they do not benefit from third-line chemotherapy, and this determines shorter OS. Conversely, in *good* classified patients, erlotinib does not worsen their clinical conditions, allowing them to take advantage from further lines, thus influencing survival. Considering that 30% of NSCLC patients are classified as *poor*, it is possible that in an unselected population, the OS difference between chemotherapy and erlotinib does not emerge. The biological rationale behind the proteomic status is currently the subject of research. Four out of the eight *m/z* peaks composing the VeriStrat *poor* profile are generated by Serum Amiloid A1 (SAA-1) and its two truncated forms (44). Moreover, in VeriStrat *poor* classified patients, higher level of a panel of anti-inflammatory proteins (haptoglobin, SAA2, SAA3, α1-antitripsyn, and α1-antichimotrypsin) was observed. SAA1 is an acute-phase protein, and it is a non-specific tumor prognostic marker (45, 46). It is induced by interleukin 1 (IL-1), interleukin 6 (IL-6), and tumor necrosis factor α (TNFα) (47). Data from literature showed that IL-6 reduced the sensitivity to erlotinib in NSCLC cells harboring EGFR mutations, due to an increased autocrine stimulation of the IL-6/gp130/signal transducer and activator of transcription 3 (STAT3) pathway (46). IL-6 activates the janus (JAK) and the Src kinases, which are responsible for the phosphorylation on the tyrosine 705 of the STAT3. Once phosphorylated, STAT3 translocates to the nucleus and activates the transcription of genes involved in cell cycle progression (cyclin D1, survivin), cell survival (B-cell lymphoma 2), angiogenesis (vascular endothelia growth factor a), and immune suppression [programed death ligand 1 (PD-L1)] (48, 49). These data suggest that the immune cells infiltrating tumor microenvironment might be the crucial determinants for influencing tumor biology, and the clinical outcome observed in VeriStrat *poor* classified patients. While erlotinib has no inhibitory effect on the stromal elements infiltrating tumor microenvironment, chemotherapy inhibits these cells, thus reducing tumor aggressiveness and prolonging survival.

Combinatorial strategies, including second-line docetaxel chemotherapy with the EGFR monoclonal antibody cetuximab, have been evaluated, with poor results. The greatest benefit was observed in those who continued previous EGFR-TKIs for ≥ 6 months (50).

## The Role of EGFR–TKIs in Patients with Squamous Histology

In the field of lung squamous cell carcinoma (LSCC), less progress has been made. Although molecular alterations in LSCC have been described, effective targeted therapies have not yet been developed (51). These potentially targetable molecular alterations include phosphoinositide 3-kinase (PIK3CA), fibroblast growth factor receptor 1 (FGFR1), or c-MET amplification and discoidin domain receptor tyrosine kinase 2 mutations, though none of these biomarkers have been validated in the clinical setting (52). The EGFR gene is commonly overexpressed in patients with LSCC (53), and two monoclonal anti-EGFR antibodies, cetuximab and necitumumab, in combination with platinum-based chemotherapy in the first-line setting, have demonstrated improved survival in phase III studies (54, 55).

Based on these data, recently, the irreversible ErbB-family inhibitor afatinib has been compared with erlotinib in the phase III Lux-Lung 8 trial, enrolling 795 squamous patients, previously progressed on platinum-based chemotherapy (Table 2) (40). The primary end-point was PFS and the primary objective was to demonstrate a 29% reduction in the risk of progression with afatinib over erlotinib. Afatinib significantly prolonged PFS and OS, health-related QoL outcomes, and symptoms control. Archived tumor tissue was collected. Six percent of the patients carried EGFR activating mutations, and another six percent harbored EGFR amplification.

Even though, based on these results, afatinib may represent an additional option for the treatment of LSCC, and it has been approved by the Food and Drug Administration (FDA) for the treatment of squamous NSCLC progressing after platinum-based chemotherapy, the new programed death 1 (PD-1) and PD-L1 inhibitors have dramatically changed the therapeutic algorithm of patients with squamous histology and represent the first therapeutic choice for second-line treatment.

## Comments and Future Perspectives

New options have emerged in the second-line armamentarium for the treatment of patients with NSCLC, including immune checkpoint inhibitors and antiangiogenic agents. The genome instability of cancer cells (56) favors the development of immunogenic clones (57). The antigen presenting cells (APC) or the dendritic cells recognize the tumor antigens, which are presented to the T cell receptors, that once activated on CD8+ T cells induce the killing of tumor cells. Inhibitory pathways have been selected to switch off the duration of the immune responses and prevent the tissue damage. Tumor cells take advantage of these inhibitory pathways to escape immune recognition and continue to proliferate. The binding of PD-1, expressed on activated T cells, tumor-infiltrating lymphocytes, and T regulatory cells, with PD-L1 or PD-L2, located on APC or tumor cells favors the T cells apoptosis and decrease cytokines production, thus modulating the immune system activation (58). Agents targeting the PD-1 axis suppress the inhibitory pathways responsible for the induction of the immune tolerance, resulting in the restoration of T cells antitumor activity. Based on the results from the phase III CheckMate-017 and CheckMate 057 trials, showing the OS improvement of the *PD-1 inhibitor* nivolumab over docetaxel in squamous and non-squamous patients, respectively, nivolumab was granted approval by the FDA and the European Medicine Agency (EMA) (59, 60). Moreover, recently, the phase III OAK study, comparing docetaxel with the *PD-L1 inhibitor* atezolizumab, showed a significant survival improvement of 27% in patients receiving atezolizumab, leading to atezolizumab FDA approval for the treatment of second-line NSCLC patients. Similarly, the phase II–III KEYNOTE-010 study, comparing the *PD-1 inhibitor* pembrolizumab with docetaxel in NSCLC patients with PD-L1 expression on at least 1% of tumor cells, showed that OS was significantly longer for pembrolizumab vs docetaxel (61). Among patients with at least 50% of tumor cells expressing PD-L1, both OS and PFS were significantly longer with pembrolizumab than docetaxel, thus determining the approval of pembrolizumab by EMA for the treatment of second-line NSCLC patients, positive for PD-L1 expression.

Another attractive therapeutic target is represented by angiogenesis, involved in the development and progression of NSCLC. Angiogenesis acts as one of the essential alterations occurring in cells during malignant transformation (56), since the delivery of oxygen and nutrients, provided by blood vessels, is required for cell survival and proliferation. Different molecules, inhibiting the angiogenic regulators, have been tested in combination with second-line chemotherapy (pemetrexed and docetaxel) in patients with NSCLC, but with disappointing results (62). Only recently, two drugs, interfering with the angiogenic pathways, nintedaninb and ramucirumab, have received the regulatory approval in association with docetaxel in the second-line setting.

Nintedanib is an oral triple angiokinase inhibitor, hindering the vascular endothelial growth factor receptor (VEGFR1–3), the FGFR1-3, the platelet-derived growth factor receptors (PDGFRα/β), fms-like tyrosine kinase 3 and members of the Src family (Src, Lyn, Lck) (63). Based on the results from the LUME Lung 1 study (64), showing a PFS improvement in patients receiving nintedanib in combination with docetaxel and a significantly prolonged OS in the subgroup of patients with adenocarcinoma, who had progressed within 9 months from the beginning of first-line treatment, the EMA approved the use of nintedanib for the treatment of locally advanced or metastatic patients with lung adenocarcinoma after platinum recurrence.

Ramucirumab is an IgG1 monoclonal antibody, targeting the extracellular domain of the VEGFR-2, thus preventing the binding of VEGF ligands and hindering receptor activation (65). When associated with docetaxel, it improves both PFS and OS (66). These clinically meaningful findings led FDA and EMA to expand the indication of ramucirumab, previously approved for the treatment of gastric cancer, to include the treatment of metastatic NSCLC.

Emerging evidence that pro-angiogenic factors have immunosuppressive activity has suggested that agents targeting angiogenesis may be potentially synergistic with immunotherapy (67–69). Data from literature indicate that VEGF influences lymphocyte trafficking, stimulates T regulatory cells and myeloid-derived suppressor cells, and inhibits T-cell development, thus favoring tumor immune escape (70–72). Moreover, it has been reported that immunotherapies can also be antiangiogenic. Different phase I trials exploring the safety and efficacy of combination regimens are currently ongoing in different types of tumors, including NSCLC.

However, based on the recent results from the Phase III KEYNOTE-024 study, showing doubling PFS and ORR in favor of pembrolizumab- vs cisplatin-based first-line chemotherapy in patients with PD-L1 expression on at least 50% of tumor cells (73), and the Phase II KEYNOTE-021 trial, demonstrating a significant PFS and ORR improvement when pembrolizumab was combined with carboplatin pemetrexed chemotehrapy, compared with chemotherapy alone (74), it is supposed that PD-1 or PD-L1 inhibitors alone or in combination with chemotherapy will become the standard of care for first-line treatment of NSCLC patients. As a consequence, clinicians will deal with new challenges for the definition of the second-line treatment algorithm.

Our knowledge on cancer immunology is not fully complete, and it is still not clear how to select those patients who benefit more from therapy with immune checkpoint inhibitors. Different studies are ongoing, and the predictive role of PD-L1 expression, evaluated by immunohistochemistry, is the focus of much research. Different PD-L1 antibodies, with different cutoff levels, have been selected according to the different PD-1 or PD-L1 inhibitors evaluated in the clinical trials. Recently, thanks to the collaboration between academy, pharmaceutical, and diagnostic companies, there has been an attempt to compare and explore the differences and the similarities between the PD-L1 diagnostic assays (75). A weak correlation was found. Other markers are under evaluation. Data from retrospective analyses indicate that tumors with a high mutational burden, abundant neoantigens, and micro-satellite high status are associated with a good response to anti-PD-1/PD-L1 therapy, but additional studies are warranted (76–79).

In conclusion, new agents have been developed and approved for the treatment of NSCLC patients without oncogene-addicted tumors, after platinum-based chemotherapy progression, thus improving the number and efficacy of therapeutic opportunities, but increasing the complexity of the therapeutic selection. Currently, the most remarkable challenge remains the lack of predictive biomarkers, able to identify which patients might gain most benefit from these agents.

## Author Contributions

The authors CL, VG, AB, MD, MV, EB, and LG have contributed equally to write this paper.

## Conflict of Interest Statement

The authors declare that the research was conducted in the absence of any commercial or financial relationships that could be construed as a potential conflict of interest.
